# Use of the behaviour change wheel to improve everyday person-centred conversations on physical activity across healthcare

**DOI:** 10.1186/s12889-022-14178-6

**Published:** 2022-09-20

**Authors:** Hamish Reid, Ralph Smith, Wilby Williamson, James Baldock, Jessica Caterson, Stefan Kluzek, Natasha Jones, Robert Copeland

**Affiliations:** 1 Moving Medicine, Faculty of Sport and Exercise Medicine, 6 Hill Square, Edinburgh, UK; 2 Advanced Wellbeing Research Centre, Sheffield Hallam University, Sheffield, UK; 3 Oxford University Hospital NHS Foundation Trust Nuffield Orthopaedic Centre, Oxford, UK; 4 School of Medicine, Trinity College Dublin, 152-160 Pearse Street, Dublin, Ireland; 5 Imperial College Healthcare NHS Trust, Praed Street, London, GB W2 1NY UK; 6 School of Medicine, University of Nottingham, Medical School, Nottingham, NG7 2UH UK

**Keywords:** Physical activity, Healthcare professional, Conversation, Behaviour change wheel

## Abstract

**Background:**

An implementation gap exists between the evidence supporting physical activity in the prevention and management of long-term medical conditions and clinical practice. Person-centred conversations, i.e. focussing on the values, preferences and aspirations of each individual, are required from healthcare professionals. However, many currently lack the capability, opportunity, and motivation to have these conversations. This study uses the Behaviour Change Wheel (BCW) to inform the development of practical and educational resources to help bridge this gap.

**Methods:**

The BCW provides a theoretical approach to enable the systematic development of behaviour change interventions. Authors followed the described eight-step process, considered results from a scoping review, consulted clinical working groups, tested and developed ideas across clinical pathways, and agreed on solutions to each stage by consensus.

**Results:**

The behavioural diagnosis identified healthcare professionals’ initiation of person-centred conversations on physical activity at all appropriate opportunities in routine medical care as a suitable primary target for interventions. Six intervention functions and five policy categories met the APEASE criteria. We mapped 17 Behavioural Change Techniques onto BCW intervention functions to define intervention strategies.

**Conclusions:**

This study uses the BCW to outline a coherent approach for intervention development to improve healthcare professionals’ frequency and quality of conversations on physical activity across clinical practice. Time-sensitive and role-specific resources might help healthcare professionals understand the focus of their intervention. Educational resources aimed at healthcare professionals and patients could have mutual benefit, should fit into existing care pathways and support professional development. A trusted information source with single-point access via the internet is likely to improve accessibility. Future evaluation of resources built and coded using this framework is required to establish the effectiveness of this approach and help improve understanding of what works to change conversations around physical activity in clinical practice.

## Introduction

In keeping with global trends, Non-Communicable Disease (NCD) dominates morbidity and mortality in the UK [1]. This position is unsustainable for the health and care system. A fundamental shift is required to move from a reactive treatment service to a proactive prevention-focused National Health Service (NHS) [2–4] that supports self-management by people living with long-term conditions [5, 6]. One way of achieving this is through a values-based, person-centred approach that enables people to effectively self-manage their long-term medical conditions with appropriate support from healthcare services [6]. Done well, up to 80% of people could self-manage their long-term medical conditions using this model of care [6].

Physical inactivity is responsible for almost 10% of the major NCDs, including heart disease, type 2 diabetes, and breast and colon cancers [7]. People living with long-term conditions are amongst the least active and stand to gain the most from even minor improvements in physical activity levels [8]. Regular contact with this hard-to-reach group makes healthcare a critical component of population approaches to addressing inactivity [9–11]. Successful integration of behaviour change strategies, which promote self-management, into routine care, including changes in healthcare professionals’ consultation behaviour, remains elusive [12]. Consequently, the management and care of people with long-term conditions are still perceived as the healthcare professional’s responsibility, rather than an active collaboration between empowered patients and a healthcare system delivering effective self-management support [5].

In keeping with the UK’s ‘Make Every Contact Count’ initiative [13], the National Institute for Health and Care Excellence (NICE) identify routine clinical conversations between healthcare professionals and patients as a vital interface to unlocking patient-driven behavioural change [11, 14]. Person-centred conversations and behaviour change are intertwined: conversations can effectively develop autonomous motivation to adopt and sustain desirable behaviours. On the other hand, the conversation itself consists of interaction behaviour that can be learned and thus changed. As part of a whole-system approach, targeting this interaction behaviour between healthcare professionals and patients may be fundamental to changing clinical practice in the NHS [5, 15].

Encouragingly, most healthcare professionals perceive conversations about physical activity to be important (ranging from 70% [16, 17] to over 90% [18–20]). Despite this, a gap exists between the proportion of times healthcare professionals perceive patients would benefit from brief opportunistic advice and the frequency with which they deliver such interventions. However, there is a disparity between perceived importance and the frequency of conversations on physical activity [12, 21] demonstrating that although healthcare professionals are receptive to the objective of physical activity promotion, a wide range of barriers exist, both individual and organisational, to putting it into practice [21, 22].

Healthcare professionals among primary and secondary care groups lack the knowledge, skills and confidence to have physical activity conversations underpinned by behaviour change theory [18, 22–33]. Whilst healthcare professionals are vocal in their support of behaviour change and self-management, they frequently minimise their ability and responsibility to deliver behavioural change work [12, 32]. Although many factors contribute to this avoidance, time concerns and previous negative experiences are powerful deterrents [22, 34]. Consequently, when healthcare professionals attempt to engage patients in conversations about change, it is often a one-sided transaction that focuses on delivering information based on the healthcare professional’s agenda for change, denying the individual the opportunity to take up more time or offer resistance [35]. Emphasis on other components of medical management reinforces this approach, such as medication review and assessment of biomarkers, which are more familiar to healthcare professionals and given greater priority by the systems in which they work. Addressing the broader context of conversations in clinical practice is essential since it is not simply a lack of time that is the issue, but prioritisation amongst the other vital components of medical management.

Collaborative discussion using evidence-based behaviour change techniques to build on a person’s thoughts about and motivation for change is more effective, better received and often more time-efficient than directive interactions [36, 37]. To help promote patient engagement and empowerment, good conversations on physical activity may use a guiding rather than directing style [34, 38]. Focus should be on the likelihood of an individual to change their own behaviour and therefore incorporate skills to emphasise autonomy such as empathic listening, fit into the available timeframe and agree individualised solutions driven by the individual’s agenda [34]. A mindset change is required to move the conversation from “what’s the matter with you” to “what matters to you”. Therefore, changing conversations on physical activity is not as simple as teaching interaction skills or telling healthcare professionals that it is important since these conversations reflect interactions that happen across an entire system.

Change in the medical workforce requires a complex combination of behaviours, decisions and interactions between healthcare professionals, patients, families and other parties. Developing an enabling culture that includes training for core skills and supporting resources to support healthcare professional behaviour change forms a central component of this complex system [39]. The effectiveness of behaviour change interventions to promote change is frequently limited by a lack of integrity with which these complex skills are delivered [40]. Whilst it is perhaps beyond the scope of practitioners to embed a comprehensive treatment fidelity framework within their clinical practice [41], an example of a practical strategy to enhance self-management would be to employ an enactment framework using video or audio recordings for patient consultations. Videos can then be reviewed and coded by the practitioner or independently by a trained third party, potentially including the use of instruments to assess specific behaviour change skills such as the implementation of Motivational Interviewing [40].

Healthcare professionals require the capability, opportunity and motivation to change their own behaviour in order to influence their patients through conversations about physical activity. We set out to understand these behavioural determinants of physical activity conversations to develop educational resources for healthcare professionals that could help them in routine clinical practice. We identified a range of high-quality pre-existing educational resources available on physical activity, such as the Swedish scientific handbook Physical Activity in the Prevention and Treatment of Disease [42]. However, we observed a lack of translation into clinical practice.

The Medical Research Council consider a theoretical basis essential for the successful development of complex interventions in healthcare [43]. The Behaviour Change Wheel (BCW) is an implementation model developed from synthesising 19 different behavioural change frameworks [44]. It provides a comprehensive structure addressing behavioural factors within nine intervention functions and seven policy categories and is advocated for use in this context by NICE [45]. The BCW helps contextualise change on an individual level and the determinants of what needs to happen to achieve organisational or system-level change. It has been used successfully to develop similar interventions improving the Capability Opportunity Motivation-Behaviour (COM-B) of healthcare professionals to deliver physical activity interventions in cancer care [46], therapeutic radiography [47], gestational diabetes [48], depression [49] and prevention of psychosis in ultra-high risk young people [50]. We believe this is the first study to utilise the COM-B framework to inform interventions focused on improving the frequency and quality of conversations on physical activity in managing long-term conditions. We are aware of studies that have used the COM-B framework to assess changes in self-reported knowledge and skills to deliver brief advice on physical activity following training [47] and studies that have explored health professionals’ practice in health care contexts such as mental health using the framework [51].

This study aims to use the BCW to analyse the behaviour of healthcare professionals and outline a coherent approach for developing interventions to improve the frequency and quality of conversations on physical activity by healthcare professionals across clinical practice.

## Methods

### Overview

The BCW outlines eight steps towards interventional design incorporating behavioural analysis using the COM-B model to understand and explore behaviour [52]. This model allowed us to draw on a parallel workstream using the COM-B model to understand the behavioural factors influencing healthcare professionals’ capability, opportunity and motivation in a national pilot developing a physical activity service in secondary care [53].

We worked through each stage of the BCW following the recommended structure summarised in Table 1 [52]. Each behaviour change component maps onto nine intervention functions (education, persuasion, incentivisation, coercion, training, enablement, modelling, environmental restructuring, and restrictions) and seven policy strategies (Environmental/social planning, communication/marketing, legislation, service provision, regulation, fiscal measures and guidelines) [44, 52]. The COM-B model recognises that behaviour is seated at the heart of this complex interacting system involving the capability (both physical and psychological), opportunity (social and physical) and motivation (reflective and automatic) of an individual or group to perform a particular behaviour [44, 52]. We expanded our behavioural analysis with the Theoretical Domains Framework (TDF), which is a framework compromising 14 domains to help identify and describe the factors influencing a particular behaviour [54, 55]. The TDF helped us achieve a deeper exploration of the barriers and facilitators to behavioural change amongst healthcare professionals and strengthen the links between the theories and techniques of behaviour change in order to anticipate and address implementation challenges [54, 56]. Following behavioural analysis and the identification of intervention options, we identified promising Behavioural Change Techniques (BCT) to inform successful intervention design [52, 57]. BCTs make up the active ingredients of interventions that allow them to be evaluated and replicated when identified in the design and evaluation of projects [45, 57–59].

**Table 1:** Developmental stages of the COM-B model

Stage 1: Understanding behaviour	Step 1	Define the problem
	Step 2	Select the target behaviour
	Step 3	Specify the target behaviour
	Step 4	Identify what needs to change
Stage 2: Identify intervention options	Step 5	Identify intervention functions
	Step 6	Identify policy categories
Stage 3: Identify content and implementation options	Step 7	Identify behaviour change techniques
	Step 8	Identify mode of delivery

### Stage 1 understanding behaviour

#### Step 1 define the problem in behavioural terms

To address step 1, over 8 weeks, the authors worked with a range of healthcare professionals working across different clinical pathways in the Active Hospital project to understand the problems faced by healthcare professionals. In addition, we, the authorship team, had weekly meetings to discuss and refine answers to the following questions defined in step 1 of the BCW:

- What is the behaviour?
- Where does the behaviour occur?
- Who is involved in performing the behaviour?

In addition, we undertook a broader scoping review [60] interrogating published evidence around physical activity conversations in clinical practice to address the question:

- What are common barriers to performing the behaviour?

#### Step 2 select the target behaviour

In step 2, we considered all factors that interventions to increase the quality and frequency of physical activity conversations could target in routine clinical care.

When deciding which behaviours to target, we considered the following four factors recommended by the BCW to inform which options are likely to be the best intervention targets:

1. The potential impact of behaviour change
2. Likelihood of the intervention leading to behavioural change
3. The impact of this behavioural change on other system components, for instance, engaging in an education program to improve skills, may increase the use of a resource to help conversations in clinical practice. The behaviour change wheel categorises this as a ‘spillover score.’
4. How easy and practical it will be to measure the target behaviour

We explored possible solutions through (1) our clinical networks across two regional meetings involving 70 multidisciplinary professionals and patients from community and hospital rheumatology and musculoskeletal services and (2) service managers, multidisciplinary leads and patients across three inpatient and one community pathway in the active hospital project [53]. We met to rate potential target areas in the four domains as unacceptable, unpromising, but worth considering, promising or very promising [52]. We made decisions by majority consensus, following discussion that considered results from the scoping review and reflected on clinical group feedback and personal experience.

#### Step 3 specify the target behaviour

In step 3, we explored the nature and characteristics of the target behaviours defined in step 2 in more detail and considered the context in which each behaviour occurs. Questions we addressed as a group included who needed to undertake the behaviour, what they needed to do, and where and when they might do it. If we were unclear about the application of the target behaviour, we spent time exploring contrasting clinical pathways to identify common characteristics. This step helped generate discrete target areas for influencing behaviour.

#### Step 4 identify what needs to change

We identified the capability, motivation and opportunity factors required to change the identified target behaviours based on the scoping review of the literature, feedback from clinical groups, departmental meetings with clinical service leaders, and discussions with inpatients involved in the active hospital project. We subsequently used the theoretical domains framework to add context to each behavioural target by working the targets through diverse clinical pathways and identifying areas of commonality. This process helped us generate discrete targets with a theoretical rationale to change practitioner behaviour successfully.

### Stage 2 identify intervention options

#### Step 5 intervention functions

In a paper exercise, we mapped COM-B components from the behavioural diagnosis onto intervention functions according to the BCW. First, we met as a group to discuss and assess each intervention function using the APEASE criteria – Affordability, Practicability, Effectiveness and cost-effectiveness, Acceptability, Side effects and safety and Equity [52]. Following this, we mapped selected intervention functions onto behavioural targets.

#### Step 6: policy categories

We identified policy categories reported in the literature and those highlighted by healthcare professionals during active hospital pathway development. As with intervention functions, we met to assess policy categories according to the APEASE criteria and mapped relevant policy categories onto behavioural domains defined in the BCW [52].

### Stage 3 identify content and implementation options

#### Step 7. Behaviour change techniques

BCTs form the active ingredients of interventions and enable coherent approaches to evaluation [61]. The BCW identifies the most frequently used BCTs by intervention function referencing ‘BCTTv1’ - a comprehensive taxonomy of 93 BCTs developed by international expert consensus [57]. We used a snowballing approach to augment BCT data from studies identified during our scoping review [60]. We identified systematic reviews reporting BCTs with promising/proven efficacy in physical activity behaviour change interventions in clinical practice [62–69]. Following this exercise, we met to map the promising BCTs onto intervention categories and identify suitable implementation strategies drawing on results from the scoping review and feedback from healthcare professionals in the active hospital clinical pathways and working groups.

#### Step 8. Mode of delivery

The final step of the BCW is to develop a delivery framework based on a recognised taxonomy of delivery modes [52]. A review of interventions that change healthcare professional behaviour [70] informed our delivery framework development, and we assessed each category using the APEASE criteria and consensus amongst ourselves.

## Results

### Stage 1 understanding behaviour

#### Step 1 define the problem in behavioural terms

We agreed on the following answers to the questions posed in step 1:

- *What is the behaviour?* Healthcare professionals initiating person-centred conversations on physical activity at all appropriate opportunities in routine medical care
- *Where does the behaviour occur?* Across the spectrum of healthcare provision for managing and treating people living with long-term medical conditions. Delivery will range from community and primary care settings to hospital inpatients in secondary and tertiary care facilities. Settings may include clinic rooms, wards, day rooms and all other environments delivering healthcare services, including remote or telehealth consultations.
- *Who is involved in performing the behaviour?* All healthcare professionals.
- *What are common barriers to performing the behaviour?* Consistent barriers to physical activity conversations in clinical practice are reported amongst a range of healthcare professionals practising in various clinical domains across numerous countries. Table 2 summarises barriers identified during the scoping review [60]:

**Table 2:** Summary of barriers to physical activity conversations in clinical practice

Barrier	Summary of the barrier
Time	Time is the most commonly reported barrier to physical activity conversations in clinical practice by all healthcare professionals [17–19, 33, 71–82]. Directly addressing time concerns is vital to improving conversations about physical activity in healthcare [22, 73, 82, 83] particularly when targeting doctors [84]. There is a delicate balance between physical activity contacts and other aspects of clinical management when delivering frontline clinical services, necessitating flexibility in any successful approach [22, 85]. Addressing both the barrier of time and appropriate allocation of resources reflects recommendations by NICE in the UK for “very brief, brief, extended brief and high intensity” behavioural change interventions [45]. Other systems reporting on approaches to physical activity consultation in practice support this approach [27, 86].
Knowledge	Healthcare professionals may lack knowledge about physical activity, and others consider the evidence base insufficiently robust [72, 81]. Those who lack knowledge ask patients about physical activity less frequently than those who consider their knowledge sufficient [76]. Although many feel comfortable giving general advice, healthcare professionals are less confident addressing detailed physical activity advice [84] and inhibited by the possibility of patients experiencing adverse events following their advice [87]. This deficit reflects inadequate training both in undergraduate and postgraduate curricula [23, 88–90].
Skills	Healthcare professionals perceive conversations around physical activity and supported self-management as important [84, 91] but those who lack confidence in their skills give physical activity advice infrequently [16, 84]. Furthermore, those who counsel patients regularly expect to be moderately or very successful, whereas those who seldom initiate physical activity discussions rarely expect them to make a difference [75]. Behaviour change skills are not traditionally taught in undergraduate medical education, and resistance to employing these skills is commonplace throughout the medical profession [32]. Attempts to integrate behaviour change skills into the undergraduate syllabus have been promising, with demonstrable improvement in understanding behaviour change principles and improved skills [32, 92]. Postgraduate training in communication skills positively impacts clinical outcomes and individual knowledge and expertise [93].
Consultation structure	The lack of a structured approach to physical activity conversations is a common barrier to effective communication amongst nurses [77]. Doctors do not appear to lack structure but are prone to taking an experimental approach to conversations, selecting behaviour change techniques arbitrarily without rationale or a coherent strategy [32].
Consultation model	Adapting routine consultations to a collaborative model will likely improve behavioural change support [5]. Healthcare professionals do not always realise when they fail to prioritise the individual’s agenda and frequently adopt a style of practice, such as diffidence or deflection, that delegitimises behaviour change talk in consultation [12]. One-sided, transactional conversations dominate this approach, failing to explore the individual’s perspective [12]. Training that focuses on building positive attitudes, self-efficacy and intentions may provide an effective strategy to address this [91, 94].
Healthcare professional-patient relationship	A lack of success, including bad experiences, during behavioural change conversations can demoralise healthcare professionals and prompt them to disengage from future attempts [32]. Accordingly, negative patient attitudes make healthcare professionals less likely to discuss physical activity with them [22, 84, 87]. Healthcare professionals perceive good interpersonal relationships as being vital in addressing behavioural change [87]. Paradoxically, positive relationships can also be a barrier to physical activity conversations since healthcare professionals may avoid them for fear of causing offence and damaging the relationship [32, 95]. Contrary to the expectation of many healthcare professionals, patients are receptive to behavioural change conversations in routine medical consultation [87]. Patients value collaborative discussion, may be resistant to a ‘preaching’ style and are most receptive when physical activity is likely to benefit their long-term medical conditions [87].
Healthcare professionals’ physical activity behaviour	Healthcare professionals’ own physical activity behaviours are a strong determinant of consultation behaviour, with less active individuals up to four times less likely to talk to people about physical activity in clinical practice [78, 96, 97].
Patient engagement	Perceived lack of motivation to change behaviour is a commonly cited barrier to physical activity conversations by healthcare professionals [21, 84]. Patients themselves welcome conversations around behaviour change [87]. Interventions encouraging patients to initiate behavioural change conversations have demonstrated success in changing healthcare professional behaviour [70, 87, 98].
System priorities	The lack of a whole system approach to integrating physical activity into routine care makes success unlikely [5, 15]. Practitioners are only likely to engage in behaviour change work if their systems value and promote it [12]. Lack of reimbursement and financial incentivisation is common but may be powerful facilitators for physical activity interventions [33, 82, 84]. It is unclear if incentivisation results in long-term change [93]. A common problem with physical activity interventions in clinical practice is that they frequently sit outside routine care pathways and lack system integration, compromising delivery [82]. Adequate resourcing, strong leadership and good communication are essential strategies to support the adoption of new processes in clinical services [70]. Systems supporting inter-professional collaboration and addressing local barriers to change positively affected care processes and patient outcomes [33, 93].
Education strategies	Despite being the most frequently used intervention [70, 99], printed education materials are likely ineffective in influencing healthcare professional behaviour if delivered passively [98, 100, 101]. The impact of educational workshops introducing physical activity is mixed [22]. Education strategies are likely to be most effective in changing healthcare professional behaviour if implemented with active strategies as part of a multifaceted intervention [93, 101–103]. Professional engagement and knowledge acquisition are improved when information is summarised and presented concisely and in an accessible format [104].
Supporting resources	A lack of information, educational resources and signposting opportunities for healthcare professionals and patients limit physical activity initiatives [88, 105, 106]. Structured and supported education, engagement and resourcing in primary care can increase physical activity contacts and have a widespread impact on organisations supporting physical activity [107]. The intention to promote physical activity is a strong predictor of consulting behaviour. Targeting professional attitudes and social norms are a promising area for success [108]. Positive messaging on best practice from subject area experts and opinion leaders promotes the uptake of best practice [93].

#### Step 2 select the target behaviour

Following analysis of the potential behavioural targets, we agreed on the initiation of conversations on physical activity by healthcare professionals as the primary target behaviour. This discrete and tangible target can be easily measured and, if achieved, is likely to prompt healthcare professionals to develop their skills. Furthermore, once initiating conversations becomes a part of healthcare professionals’ routine consulting practice, they are likely to influence others’ practice positively. Table 3 outlines the behavioural analysis.

**Table 3:** Prioritising behavioural interventions

Potential target areas	Potential behavioural targets	Impact of behaviour change	Likelihood of changing behaviour	Spillover score	Measurement score	Selection
Healthcare professionals	Initiating physical activity conversations routinely	very promising	promising	very promising	promising	Primary target
	Following a counselling protocol to help structure consultations	promising	promising	Promising	very promising	Secondary target
	increasing own physical activity levels	promising	promising	very promising	promising	unfeasible
	engaging in education and training to improve skills and knowledge	promising	very promising	Promising	very promising	Secondary target
Patient behaviour	Prompting patient initiation of conversation	very promising	very promising	Promising	promising	Secondary target
	Independent efforts to increase physical activity	unpromising, but worth considering	very promising	Promising	unpromising, but worth considering	Unfeasible
Environment	protecting dedicated time for physical activity conversations	unpromising, but worth considering	very promising	promising	promising	Unfeasible
	peer support in the workplace for physical activity promotion	promising	promising	very promising	unpromising, but worth considering	Secondary target
	service expectations	very promising	very promising	very promising	promising	Unfeasible
Systems	using a resource to help structure conversations	Promising	promising	very promising	unpromising, but worth considering	Secondary target
	visiting a resource to learn more	promising	very promising	promising	very promising	Secondary target
	responding to system requirements for physical activity conversations	very promising	very promising	very promising	promising	Unfeasible

#### Step 3 specify the target behaviour

After specifying the primary and secondary target populations and behaviours, we specified the behavioural target regarding who, what, where and when the behaviour is performed. This helped us generate the discrete target areas for influencing behaviour outlined in Table 4.

**Table 4:** Specifying target behaviours

Selected behavioural targets	Behavioural specifications			
	Who	What	Where	When
Having conversations routinely about physical activity	Healthcare professionals	Talking to patients about physical activity as part of routine care	All healthcare environments	During routine clinical care
Using a resource to improve conversations	Healthcare professionals	Access a resource to provide them with the information they require to have good quality conversations on physical activity	It needs to be available in all places of practice, so a digital resource is likely to be best	Use resources during routine clinical conversations
Engaging in education and training to improve skills and knowledge	Healthcare professionals	Undertake independent learning and CPD in behavioural change counselling	Place of work or during independent study time, which could be in various locations, including libraries and at home	During the time set aside by the individual and/or employer for professional development
Patient initiation of conversation	People attending healthcare appointments	Ask their healthcare professional about the role of physical activity in managing their health	Healthcare contacts	Routine medical care
Peer support in the workplace	Healthcare professionals and support staff	Communicate with colleagues about physical activity as an essential part of clinical care	Healthcare environments	During meetings, case discussions, practise updates
Use a practical structure for consultations	Healthcare professionals	Use a timeframe-based template to guide conversations	Healthcare environments	During conversations with patients in routine care

#### Step 4 identify what needs to change

The behavioural diagnosis identified barriers and facilitators to all six core components of the COM-B model. Expanding each domain using the theoretical domains framework helped us define tangible targets for intervention design, as demonstrated in Table 5.

**Table 5:** Behavioral diagnosis and theoretical domain mapping

Behavioral targets *[E] – Enablers [B] - barriers*		Identifying what needs to change using the theoretical domains framework	
Capability - Physical	[E] Healthcare professionals are more likely to have physical activity consultations if they are active themselves [75, 78, 99, 100] [E] Training on how to use resources will improve utility [21, 35, 79]	**Physical skills**	Information and resources supporting the integration of activity into daily life should be as applicable to healthcare professionals as patients [78, 99] Supporting resources should include training capability on the skills required for good quality conversations about physical activity [101–104]
Capability -Psychological	Psychological barriers to engagement include [35, 75, 79, 105]: [B] Uncertainty around advice for specific conditions [B] The perception that other lifestyle factors are easier to address and more important [B] Physical activity and behavioral change education is generally limited [46, 106–111]	**Knowledge**	Resources should address the widely reported deficit in knowledge on physical activity in the management of long term conditions [21, 31, 35, 99]
		**Cognitive and interpersonal skills**	Traditional transactional medical consultation models do not transfer well into behavioral change discussions, so solutions need to promote and support person-centered behavioral change conversations [33, 63, 69, 103, 105, 112]
		**Memory attention and decision processes**	Clinical staff already have many tasks and objectives during a consultation. Wherever possible, interventions should be structured in such a way that they can fit naturally into clinical workstreams in a time-sensitive fashion [35, 113]
Opportunity - Physical	[B] Actual and perceived lack of time are fundamental barriers to physical activity conversations [21, 35, 74, 75, 100, 114] [E] Computers are fundamental to healthcare delivery, so freely available online solutions are likely to increase accessibility [E] Signposting of resources and support structures need to be clear [21, 101, 102, 104, 115] [E] Printable elements such as campaign posters and patient leaflets should be easily and freely available [60, 79] [E] Pathway support will benefit from tools to facilitate physical activity behavioral change [21, 100, 115]	**Environmental context and resources**	Lack or perceived lack of time must be addressed upfront [20, 27, 66, 100] Busy work environments and service delivery pressures often impact continual professional development (CPD), including taking on new knowledge and learning new skills. Successful training solutions will support the delivery of routine care and CPD requirements [20, 27, 66, 100] Few healthcare environments are activity permissive. Resources for system support and promoting cultural change are likely to help clinical staff be more active [99] Clinical staff are not clear on where they can get good quality physical activity materials and resources. Memorable and easily accessible signposting resources are required [102, 116, 117]
Opportunity - Social	[E] Involving professional bodies and disease area specialists in the design and development of solutions supporting clinical practice will improve credibility [100, 118] [E] Peer support from other healthcare professionals and the workplace environment increases opportunity [100, 118] [E] Peer-group supported online learning and ambassador network has the potential to improve physical activity delivery by developing lasting social opportunities [100] [E] Prompts coming from patients themselves will encourage HCPs to reflect and prioritize physical activity in consultation [112]	**Social influences**	Physical activity is currently a lower priority in routine clinical practice than other behavioral components such as smoking, drinking alcohol and eating [75]. As a result, clinical pathways (except for a few notable exceptions, such as cardiac rehabilitation) do not promote normative peer group behaviors around physical activity conversations. Resources targeting specific conditions may help to clarify the role of physical activity in general care pathways [83]
Motivation - Reflective	[E] Healthcare professionals believe that conversations about physical activity are important [21, 105] [B] Many healthcare professionals recognize physical activity as important but do not feel they have the knowledge or confidence to counsel patients effectively [21, 31, 35, 99, 105] [B] Healthcare professionals frequently avoid talking about physical activity for fear of provoking resistance, so it is likely building confidence in techniques that avoid generating resistance behaviors and promote positive experiences will improve motivation [21, 66, 75, 78] [E] Evidence should be published by a trustworthy source as healthcare professionals consider this essential to believing it. Ideally the evidence base will also be presented in an easily accessible format to improve engagement [119] [B] Interventions are required to help build clinician self-efficacy [35, 79] [B] Perceived lack of success	**Social/ professional role & identity**	Supporting patients to lead physically active lives lies at the heart of healthcare and therefore is a responsibility of all healthcare professionals. Identifying role-specific intervention strategies and re-enforcing when and where it is a priority for healthcare professionals will help address this [11, 35, 75, 116, 120] Fostering a leadership culture amongst colleagues by giving early adopters resources and confidence in their knowledge can help drive change [121, 122]
		**Beliefs about capabilities**	Self-efficacy is a relevant intervention target since the confidence of a healthcare professional in their ability to impact upon patient behavior and trust in support from the healthcare system is associated with improved frequency and quality of physical activity conversations [101–104] Those who report physical activity behavior change conversations negatively seldom initiate them. Resources helping build self-efficacy through reflective practice can help improve this [11, 21, 35, 75, 79, 100]
		**Optimism**	Healthcare professionals who discuss physical activity frequently with patients generally feel confident they will have an impact. Empowering these individuals to influence their colleagues who do not frequently discuss physical activity and do not believe they will make a difference may help improve optimism [11, 35, 75, 100]
		**Intentions**	Healthcare professionals do not always see it as their role to influence physical activity, but a system-wide approach requires appropriate intervention at all opportunities. Solutions should be flexible enough to help clinicians in all roles make a constructive contribution [35, 75, 116, 120]
		**Goals**	Clinical delivery targets and guidelines drive goals in clinical practise. Facility to generate physical activity metrics may assist this service development [27, 101] Healthcare professionals benefit from individual goals for professional development when developing new skills
		**Beliefs about consequences**	Healthcare professionals who believe their conversations on physical activity are well-received talk more frequently about physical activity. Patient-led prompts can help to improve this [112]
Motivation - Automatic	[E] Conversations on physical activity around the management of long-term conditions should become habitual for healthcare professionals. Contributing to this is confidence in conversations about physical activity, demand from patients, the behavior of peers and demands of clinical practise [E] Systematic prompts in medical records systems are beneficial for building habits [E] Solutions should meet expected standards of practice, guidelines and best-practise management strategies [123]	**Reinforcement**	Promoting patient initiation of conversations on physical activity and peer group discussions/learning incentivizes conversations and reinforces the importance with healthcare professionals [119]
		**Emotion**	Physical activity behavior change conversations that go badly can lead to patients becoming upset, particularly if they feel judged. Healthcare professionals who have had such unpleasant experiences frequently avoid future conversations in the fear they may end up the same way [66, 78] Training in counselling skills throughout undergraduate and postgraduate education can help improve this [102, 124]

### Stage 2 identify intervention options

#### Step 5 intervention functions

In total, we selected six out of nine intervention functions. We deemed incentivisation not affordable or practical, coercion not practical or acceptable, and restriction not practical for implementation in general clinical environments. Table 6 maps intervention functions onto behavioural targets.

**Table 6:** Selecting intervention functions

Intervention function	Does intervention meet APEASE criteria?	Behavioural target
Education	yes	psychological capability and reflective motivation
Persuasion	yes	automatic and reflective motivation
Incentivisation	no	*not affordable or practical*
Coercion	no	*not practical or acceptable*
Training	yes	physical and psychological capability
Restriction	no	*not practical*
Environmental restructuring	yes	physical and social opportunity, automatic motivation
Modelling	yes	automatic motivation
Enablement	yes	physical and psychological capability, physical opportunity, automatic motivation

#### Step 6: policy categories

We selected 5 out of 7 policy categories, although regulation relies on external bodies for practicability. Despite this, we agreed to include regulation because service leaders in clinical pathways we were developing in the active hospital project were keen to change the systems regulating practice by healthcare professionals in their pathways, for instance, through the electronic records system. Fiscal and legislative policy categories were deemed impracticable and unacceptable. Table 7 outlines an assessment of each policy category and examples of potential delivery mechanisms for a resource to support conversations on physical activity.

**Table 7:** Identifying policy categories to support intervention delivery

Policy Category	APEASE criteria met?	Behavioural domain	Description of potential delivery mechanisms
Communication/ Marketing	Yes	Capability - Psychological	Campaign materials. Video. Communication from professional bodies. Printable materials, including patient information, posters and digital prompts
		Motivation - Reflective	Behaviour monitoring tools, goal setting resources and workbook. Print and digital resources, including the evidence base on physical activity in managing specific conditions. Quotes from patients and influential professionals
		Motivation - Automatic	Demonstrate peer approval and encourage role modelling through resources, supporting campaigns and ambassador network. Develop video resources to model good quality conversations. Include patients, families and friends in campaigns to prompt patient-driven consultation on physical activity
Environmental/ social planning	Yes. Effectiveness relies on successful communication and dissemination	Capability - Physical	Design flexible resources to support conversations no matter how much time is available. Support with campaign and training resources to promote skill acquisition
		Capability - Psychological	Support delivery of care in existing pathway models. Supporting materials aimed at promoting discussion and awareness
		Opportunity - Physical	Make available free and online for use on desktop, tablet or mobile device
		Motivation - Automatic	Social networking with ambassador programme, press & media and educational network
Fiscal	No. Not practicable or acceptable		Nil
Guidelines	Yes	Capability - Physical	NICE guidelines recommend supporting outputs on delivery of physical activity and behavioural change, as well as disease-specific pathways and guidelines for best practice. Make resources available in regular IT systems, support with campaign and other resources, offline capability
		Capability - Psychological	Make healthcare professionals aware of guidelines recommending physical activity as part of routine disease management in their specialist area.
		Opportunity - Physical	An information dissemination plan including messages from professional bodies and trusted sources
		Motivation - Automatic	Draw attention to and provide education on current standards and guidelines. Seek endorsement from respected professional organisations
Legislation	No. Not practical		nil
Regulation	Yes, although practicability relies on extrinsic regulation of best practice	Capability - Psychological	As expected by best practice standards, educate on the importance of including physical activity contacts as a regular part of routine clinical care. Reassure healthcare professionals on the effectiveness and cost-effectiveness. Make these resources available for pathway leaders to implement locally
		Motivation - Reflective	Design resources to support mandatory components of practice such as care pathways recommending brief advice on lifestyle factors or assessment of physical activity levels
Service Provision	Yes	Capability - Physical	Structured guidance on how to use a resource during regular service delivery to facilitate ongoing learning during routine clinical practice
		Capability - Psychological	Promote role modelling through individual clinical practice, sharing the experience of others through online peer support.
		Opportunity - Physical	Directly address the time constraints of clinical practice by providing solutions based on available time. Make available resources promoting patients to initiate physical activity discussions such as posters and information leaflets for waiting rooms
		Opportunity -Social	Facilitate healthcare professionals developing networks to share and promote good practise around physical activity in clinical practice. Make slidesets available for peer group learning sessions
		Motivation - Reflective	Customise resources for each speciality to meet pathway and guidance requirements
		Motivation - Automatic	Frame concerns and barriers to physical activity in a conversational framework to support time-responsive learning and development during service provision. Base any ‘how-to-use’ resources on a clinical consultation model to augment existing practice.

### Stage 3 identify content and implementation options

#### Step 7 behaviour change techniques

We identified 17 promising BCTs and mapped these onto BCW intervention functions, as demonstrated in Table 8. Selected BCTs included 1. Goals and planning, 2. Feedback and monitoring, 3. Social support, 4. Shaping knowledge, 5. Natural consequences, 9. Comparison of outcomes, 12. Antecedents, and 15. Self-belief.

**Table 8:** Mapping BCTs to COM-B component, intervention functions and implementation strategy

Behavior change technique (BCTv1)		Motivation - Automatic	Motivation - Reflective	Capability - Psychological	Capability - Physical	Opportunity - Physical	Opportunity - Social	Implementation strategy
1.1 [45, 55–57]	Goal setting (behavior)	Enablement						Goal setting plans and workbooks to facilitate goal setting in practice
1.2^55–57,59^	Problem-solving	Enablement		Enablement				Evidence-based practical solutions to problems that come up in consultation, such as how to introduce the topic and address common concerns
1.3 [45, 57]	Goal setting (outcome)				Enablement			Provide resources to support collaborative goal setting
1.4 [55, 56, 58]	Action planning				Enablement			Provide resources to support collaborative action planning
1.6 [55]	Discrepancy between current behavior and goal	Enablement			Enablement			Provide examples outlining the difference between successful and unsuccessful behavioral change conversations on physical activity and offer supporting education opportunities to address common problems recognized by clinicians Provide a mechanism to assess physical activity levels for comparison to national recommendations Identify disease-specific requirements of best practice
2.3^55–57,59^	Self-monitoring of behavior		Education	Enablement		Enablement		Encourage reflective practice on the frequency of physical activity conversations Provide self-monitoring tools and materials for healthcare professionals to use
4.1 [45, 55, 56, 58]	Instruction on how to perform a behavior			Training	Training			Demonstration on how to use physical activity resources. Include direction and supporting training capability on how to improve conversations on physical activity
5.1 [45, 55, 58]	Information about health consequences		Education and persuasion	Education				Provide education on the benefits of physical activity on the prevention and treatment of disease Present comparably to other medical resources and report their evidence base Customize resources by disease area and focus on symptomatic benefits
5.3 [55, 56]	Information about social and environmental consequences		Education and persuasion	Education				Give information on the global burden of physical inactivity and the role of healthcare in addressing this Demonstrate the impact of physical activity counselling on general wellbeing and crossover benefits for conversations with patients and colleagues regarding other healthcare issues Include quotes from patients and other professionals
6.2 [45, 58]	Social comparison		Persuasion					Develop assets relaying positive messages around the importance of physical activity conversations from patients, influential clinicians and professional bodies
6.1 [45, 56, 58]	Demonstration of the behavior	Modelling		Training	Training			Provide supporting training materials demonstrating real-life conversational skills between healthcare professionals and members of the public in routine clinical practice Identify things to include and things to avoid when talking about physical activity to promote positive interactions
6.3	Information about others’ approval	Persuasion	Education, Persuasion	Education				Develop and disseminate in collaboration with affiliated professional bodies to provide credibility and alternate avenues for education Facilitate the development of an ambassador network of engaged and like-minded individuals to take on local leadership roles Include quotes from patients and other professionals
7.1 [45, 58]	Prompts/cues	Environmental restructuring				Environmental restructuring	Environmental restructuring	Provide resources such as leaflets, posters and digital assets, for instance, screensavers and email for display to improve visibility, awareness and prompt both healthcare professionals and members of the public to initiate physical activity conversations Align with mandatory components of incentivized practise such as brief advice in established guidelines and care pathways
8.1 [45, 56, 58]	Behavioral practice/ rehearsal			Training				Advocate reflective practice and provide access to training resources
9.1 [58]	Credible source	Persuasion	Persuasion					Develop a trusted and recognizable source of information on physical activity in clinical practice Peer approval and communication through collaboration and development with professional bodies
9.2 [58]	Pros and cons			Enablement				Explain the pros and cons of person-centered approaches to conversations on physical activity, citing relevant literature and fundamental concepts of behavioral change
12.1	Restructuring the physical environment					Enablement		Form a recognizable an open access, internet-based single point of access to disease-specific information on physical activity in the management of long-term conditions for healthcare professionals to access wherever they work
12.2 [56]	Restructuring the social environment	Environmental restructuring and enablement				Environmental restructuring	Environmental restructuring	Help define social norms around physical activity behaviors Offer time-saving options for busy clinicians Encourage patient-led initiation of physical activity conversations and give access to educational resources that colleagues can share Provide an online community to support learning and facilitate sharing of good practice across workplaces
12.5 [55–57]	Adding objects to the environment	Environmental restructuring		Enablement	Enablement	Environmental restructuring and enablement		Provide resources to be displayed, for instance, in waiting areas, to prompt discussion and awareness amongst members of the public Provide an online forum for interprofessional communication Make all resources freely available online
13.1	Identification of self as a role model	Persuasion	Persuasion	Enablement				Share information on the importance of clinician behavior on influencing good practice amongst their patients and colleagues
15.3	Focus on past success		Persuasion					Provide resources and educational strategies to help clinicians reflect on positive conversations they may have had with patients about physical activity in the past and suggest theoretical reasons why they may have worked to help build self-efficacy
15.4 [45, 55]	Self-talk				Training			Encourage users to rehearse their conversational approach with patients

#### Step 8. Mode of delivery

We focused on population-level delivery approaches to make a resource accessible to as many healthcare professionals as possible. In addition, Digital channels predominate to make the project affordable and broaden its reach. Table 9 outlines our review of the BCW delivery framework.

**Table 9:** Defining the intervention delivery framework using APEASE criteria

Mode of delivery				APEASE criteria met?
Face to face	Individual			Not practicable
	Group			Conference presentations and workshops are suitable outlets for professional dissemination. In-person training is preferable.
Distance	Population-level	Broadcast media	TV.	Direct TV coverage, including advertising, is not affordable. However, press releases and project events promoted through professional groups and collaborating partners may lead to TV exposure.
			Radio	Primary radio coverage through advertising is not affordable. However, press releases and project events promoted through professional groups and collaborating partners may lead to radio exposure.
		Outdoor media	Billboard	Not affordable
			Poster	Freely available digital posters online. Effectiveness relies on uptake by collaborators and stakeholders for printing and usage of posters and campaign materials
		Print media	Newspaper	Advertising in newspapers is not affordable. However, journalists may carry stories related to press releases, and professionals may promote a physical activity intervention in health-related coverage
			Leaflet	Patient-facing digital leaflets freely available online, so effectiveness relies on local usage of posters and campaign materials
		Digital media	Internet	The internet offers the primary route for dissemination of an open-access resource available in healthcare professionals’ workplaces
			Mobile phone app	Optimise internet resources for use on devices and mobile platforms
			Social media	Social media can engage established professional networks through resource collaborations and influential drivers of social media content around physical activity and health. Over time a digital presence can be established
	Individual	Phone	Phone helpline	Not practicable or affordable
			Text message	Not practicable or equitable
		Individually accessed computer programme		Supporting training programmes may be accessed online, and resources made freely available

## Discussion

This study uses the BCW to outline a coherent approach for intervention development to improve the frequency and quality of conversations on physical activity by healthcare professionals managing long-term conditions. Time-sensitive and role-specific resources will help healthcare professionals understand the focus of their intervention. Educational resources aimed at healthcare professionals and patients will have mutual benefit, should fit into existing care pathways and support professional development. A trusted information source with single-point access via the internet will improve accessibility and provide an ideal delivery mechanism for a wide range of resources, including an avenue for distributing free promotional information.

Our concurrent clinical activity in the development of three inpatient and one community clinical physical activity pathway in the Active Hospital pilot provided an ideal environment to explore and test promising ideas from published literature. We balanced our behavioural analysis across community and hospital environments. However, we recognise there is a risk of a bias toward understanding the implementation landscape in a hospital environment, potentially limiting the applicability of our findings to interventions in other settings, such as primary care. Although our clinical backgrounds positively impact the clinical relevance of this study, the quality of our work is potentially limited by the lack of a robust academic background in behaviour change amongst the healthcare professionals in our team. This impacted some of our decision-making; for instance, we included modifying patient behaviour as a secondary behavioural target due to promising literature suggesting the benefits from this approach without behavioural analysis of patients themselves. We identified the risk of our academic limitations at the outset, which informed our decision to use the BCW due to its straightforward and step-wise guidance. As others have reported, we discovered that following the BCW system is an exacting challenge [46]. Systematically following all steps was laborious and time-consuming, but it ensured consideration of all components of effective behaviour change [109, 110]. We followed the model diligently; for instance, we spent time defining the primary behavioural target despite other authors deeming this unnecessary [46]. As we subsequently progressed through the stages, we found that defining the target was a great benefit as it helped us maintain focus on changing the consulting behaviour of healthcare professionals rather than the physical activity behaviour of their patients. In some areas, we found the scope of the challenge exceeded our resources and looked to previously published evidence for guidance. For example, we narrowed down BCT choice by identifying promising BCTs in the published literature. However, failing to fully consider and explore all 93 BCTs on their individual merits may mean we missed effective BCTs whose use may be novel in this area.

A growing body of evidence demonstrates the potential of time-efficient behavioural change approaches in clinical practice [82, 111, 112]. People living with long-term conditions value and welcome behavioural change support on physical activity from healthcare professionals [69, 113]. However, traditional transactional models of clinical consultation offer an over-simplistic and ineffective approach to encouraging behavioural change. This model of medicine, established over generations, is not without limitations when considering straightforward prescription, such as antihypertensives [114] or even major surgery, such as solid organ transplant [115]. Conversations to support behavioural change should start with the individual and consider personal choice, circumstance and behavioural context, suggesting the traditional consultation model of ‘diagnose and treat’ requires a rethink [116]. This study confirms that successful resources should consider individual preference, circumstance, behavioural context, and system constraints such as appointment length to support physical activity conversations effectively.

Whilst education and training alone are insufficient to change healthcare professional behaviour, embedding education and training opportunities into a practical structure to support routine practice improves practitioner engagement and increases the likelihood of behavioural change [117]. Healthcare systems must value and promote such an intervention as the prevailing professional, and organisational culture may be most influential in changing practitioner behaviour [70, 102, 118]. Success may be when healthcare professionals habitually include person-centred physical activity conversations in their practice. Given the range of competing interests on their time, automisation of their behaviour through habit formation is likely to free up cognitive capacity [119]. Although automaticity has been successfully targeted in simple healthcare tasks such as hand washing, complex tasks such as physical activity conversations appear less conducive to habit-forming [120]. However, targeting specific behavioural components may be a way around this challenge [119]. For instance, system support can influence habit formation through intervention such as integrating prompts in computer systems or clinical pathways.

As well as prompting habit formation, building educational resources into routine care by supporting real-time decision-making and providing point-of-care prompts for best practice can enhance professional development [102]. Such education strategies have a more significant impact when derived from influential opinion leaders [70, 98]. Developing strategies informed by likely barriers and facilitators of behavioural change to translate research findings into clinical practice can further enhance effectiveness [121, 122]. Digital approaches successfully support clinical decision-making and the delivery of preventative care [98, 102, 123, 124]. Delivery via the internet supports several behavioural domains identified in this study and is a simple way to deliver a scalable and cost-effective intervention [122, 125].

Future research may include understanding how to leverage the influence of patients on healthcare professional behaviour and improve habituation within complex communication skills. Greater understanding is required of how healthcare systems and the professionals within them can best balance the fundamental medical requirements of long-term condition management with individualised and person-centred behavioural change support. Designing interventions with evaluation in mind is critical to help understand the optimal approach to increasing the frequency and quality of conversations on physical activity across clinical practice. To this end, the findings of this study have informed the development of a hybrid online resource combining educational material with conversational guidance coded with BCTs to support evaluation [44, 59]. We encourage independent evaluation of the resources at www.movingmedicine.ac.uk and call upon researchers to focus on improving our understanding of what works to improve conversations on physical activity across clinical practice.

## Conclusion

We have iteratively developed a framework using the BCW to improve healthcare professionals’ capability, opportunity and motivation to have person-centred conversations on physical activity. The framework is grounded in the priorities of busy healthcare professionals addressing a range of barriers, including time, knowledge, skills and system support. At the heart of a successful intervention lies the principles of person-centred care and an approach that may be unfamiliar to healthcare professionals trained in a didactic consultation style. Resources need to be time-sensitive and role-specific, whilst educational resources aimed at healthcare professionals and patients will have mutual benefit, should fit into existing care pathways and support professional development. A trusted information source with single-point access via the internet will improve accessibility and provide an acceptable delivery mechanism for a wide range of resources. All healthcare team members have a role in delivering constructive physical activity support.

Building practical resources based on this framework will improve efficacy, integrate the principles of behaviour change and provide a platform to inform future research and develop clinical physical activity services. Therefore, we encourage open evaluation of resources built using this framework to help improve understanding and implementation of what works.

## Data Availability

All the datasets generated and/or analysed during this framework analysis are available in the Centre for Open Science OSF repository at https://osf.io/muxdy/?view_only=223e9f46226245e3a4ff2882b061e41a

## Abbreviations

BCT: Behaviour Change Technique
BCW: Behaviour Change Wheel
FSEM: Faculty of Sport and Exercise Medicine
NHS: National Health Service
NICE: National Institute for Health and Care Excellence
NCD: Non-Communicable Disease
UK: United Kingdom

## References

[1] Bennett JE, Stevens GA, Mathers CD, Bonita R, Rehm J, Kruk ME (2018). NCD Countdown 2030: worldwide trends in non-communicable disease mortality and progress towards sustainable development goal target 3.4. Lancet.

[2] Ham C (2017). Next steps on the NHS five year forward view. BMJ.

[3] NHS (2019). The NHS Long Term Plan.

[4] Darzi A. The Lord Darzi review of health and care interim report. London; 2018. www.ippr.org. Accessed 30 Jul 2018

[5] Coulter A, Roberts S, Dixon A (2013). Delivering better services for people with long-term conditions.

[6] Wagner EH, Austin BT, Davis C, Hindmarsh M, Schaefer J, Bonomi A (2001). Improving chronic illness care: translating evidence into action. Health Aff.

[7] Lee IM, Shiroma EJ, Lobelo F, Puska P, Blair SN, Katzmarzyk PT (2012). Effect of physical inactivity on major non-communicable diseases worldwide: an analysis of burden of disease and life expectancy. Lancet.

[8] Woodcock J, Franco OH, Orsini N, Roberts I (2011). Non-vigorous physical activity and all-cause mortality: systematic review and meta-analysis of cohort studies. Int J Epidemiol.

[9] The International Society for Physical Activity and Health ISPAH (2020). International Society for Physical Activity and Health’s eight investments that work for physical activity.

[10] Weiler R, Feldschreiber P, Stamatakis E (2012). Medicolegal neglect? the case for physical activity promotion and Exercise Medicine.

[11] NICE (2015). Physical activity : encouraging activity in all people in contact with the NHS.

[12] Hunter C, Chew-Graham CA, Langer S, Drinkwater J, Stenhoff A, Guthrie EA (2015). “I wouldn’t push that further because I don’t want to lose her”: a multiperspective qualitative study of behaviour change for long-term conditions in primary care. Health Expect.

[13] Health Education England (2018). Making every contact count (MECC).

[14] NICE (2013). Physical activity: brief advice for adults in primary care primary care. Natl Inst Heal Care Excell Public Heal Guidel.

[15] Speake H, Copeland RJ, Till SH, Breckon JD, Haake S, Hart O (2016). Embedding physical activity in the heart of the NHS: the need for a whole-system approach. Sport Med.

[16] Cho H-JJ, Sunwoo S, Song Y-MM (2003). Attitudes and reported practices of Korean primary care physicians for health promotion. J Korean Med Sci.

[17] Ribera APA (2005). McKenna J, Riddoch C, of CR-TEJ, 2005 U. attitudes and practices of physicians and nurses regarding physical activity promotion in the Catalan primary health-care system. Eur J Pub Health.

[18] Douglas F, Torrance N, van Teijlingen E, Meloni S, Kerr A (2006). Primary care staff’s views and experiences related to routinely advising patients about physical activity. A questionnaire survey. BMC Public Health.

[19] Lawlor D, Keen S, Practice RN-F (1999). Increasing population levels of physical activity through primary care: GPs’ knowledge, attitudes and self-reported practice. Fam Pract.

[20] Reed B, Jensen J (1991). Preventive DG-A journal of, 1991 U. physicians and exercise promotion. Am J Prev Med.

[21] Keyworth C, Epton T, Goldthorpe J, Calam R, Armitage CJ (2018). Are healthcare professionals delivering opportunistic behaviour change interventions? A multi-professional survey of engagement with public health policy. Implement Sci.

[22] Huijg JM, van der Zouwe N, Crone MR, Verheijden MW, Middelkoop BJCC, Gebhardt WA (2015). Factors influencing primary health care professionals’ physical activity promotion behaviors: a systematic review. Int J Behav Med.

[23] Dacey ML, Kennedy MA, Polak R, Phillips EM (2014). Physical activity counseling in medical school education: a systematic review. Med Educ Online.

[24] Levy MD, Loy L, Zatz LY (2014). Policy approach to nutrition and physical activity education in health care professional training. Am J Clin Nutr.

[25] Kordi R, Moghadam N, Rostami M. Sports and exercise medicine in undergraduate medical curricula in developing countries: a long path ahead. Med Educ Online. 2011;16. 10.3402/meo.v16i0.5962. 10.3402/meo.v16i0.5962 PMC3040663 21350601

[26] Joy EL, Blair SN, McBride P, Sallis R (2013). Physical activity counselling in sports medicine: a call to action. Br J Sports Med.

[27] Sallis R, Franklin B, Joy L, Ross R, Sabgir D, Stone J (2015). Strategies for promoting physical activity in clinical practice. Prog Cardiovasc Dis.

[28] Douglas F, van Teijlingen E, Torrance N, Fearn P, Kerr A, Meloni S (2006). Promoting physical activity in primary care settings: health visitors’ and practice nurses’ views and experiences. J Adv Nurs.

[29] Knox ECL, Musson H, Adams EJ (2015). Knowledge of physical activity recommendations in adults employed in England: associations with individual and workplace-related predictors. Int J Behav Nutr Phys Act.

[30] Knox ECL, Esliger DW, Biddle SJH, Sherar LB (2013). Lack of knowledge of physical activity guidelines: can physical activity promotion campaigns do better?. BMJ Open.

[31] Chatterjee R, Chapman T, Brannan MG, Varney J (2017). GPs’ knowledge, use, and confidence in national physical activity and health guidelines and tools: a questionnaire-based survey of general practice in England. Br J Gen Pract.

[32] Chisholm A, Hart J, Lam V, Peters S (2012). Current challenges of behavior change talk for medical professionals and trainees. Patient Educ Couns.

[33] Albert FA, Crowe MJ, Malau-Aduli AEO, Malau-Aduli BS (2020). Physical activity promotion: a systematic review of the perceptions of healthcare professionals. Int J Environ Res Public Health.

[34] Rollnick S, Butler CC, McCambridge J, Kinnersley P, Elwyn G, Resnicow K (2005). Consultations about changing behaviour. BMJ Br Med J.

[35] Laverack G (2017). The challenge of the ‘art and science’ of health promotion. Challenges.

[36] Hillsdon M, Thorogood M, White I, Foster C (2002). Advising people to take more exercise is ineffective: a randomized controlled trial of physical activity promotion in primary care. Int J Epidemiol.

[37] Gagliardi AR, Faulkner G, Ciliska D, Hicks A (2015). Factors contributing to the effectiveness of physical activity counselling in primary care: a realist systematic review. Patient Educ Couns.

[38] Lundahl B, Moleni T, Burke BL, Butters R, Tollefson D, Butler C (2013). Motivational interviewing in medical care settings: a systematic review and meta-analysis of randomized controlled trials. Patient Educ Couns.

[39] Health Education England, Skills for Health and S for C (2017). Person-Centred Approaches: Empowering people in their lives and communities to enable an upgrade in prevention, wellbeing, health, care and support.

[40] Moyers TB, Rowell LN, Manuel JK, Ernst D, Houck JM (2016). The motivational interviewing treatment integrity code (MITI 4): rationale, preliminary reliability and validity. J Subst Abus Treat.

[41] Borrelli B (2011). The assessment, monitoring, and enhancement of treatment Fidelity in public health clinical trials. J Public Health Dent.

[42] Public Health Agency of Sweden. On behalf of the EUPAP Consortium. EUPAP FYSS-short - physical activity in the prevention and treatment of disease. Sweden; 2019. www.fhi.se

[43] Skivington K, Matthews L, Simpson SA, Craig P, Baird J, Blazeby JM, et al. A new framework for developing and evaluating complex interventions: update of Medical Research Council guidance. BMJ. 2021;374. 10.1136/BMJ.N2061. 10.1136/bmj.n2061 PMC8482308 34593508

[44] Michie S, van Stralen MM, West R (2011). The behaviour change wheel: a new method for characterising and designing behaviour change interventions. Implement Sci.

[45] NICE. Behaviour change: individual approaches | guidance and guidelines | NICE. Natl Inst Heal Care Excell. 2014:49 https://www.nice.org.uk/guidance/ph49. Accessed 15 Feb 2018.

[46] Webb J, Foster J, Poulter E (2016). Increasing the frequency of physical activity very brief advice for cancer patients. Development of an intervention using the behaviour change wheel. Public Health.

[47] Pallin ND, Webb J, Brown L, Woznitza N, Stewart-Lord A, Charlesworth L (2022). Online training resources to aid therapeutic radiographers in engaging in conversations about physical activity and diet: a mixed methods study. Radiography.

[48] Smith R, Michalopoulou M, Reid H, Riches SP, Wango YN, Kenworthy Y (2022). Applying the behaviour change wheel to develop a smartphone application ‘stay-active’ to increase physical activity in women with gestational diabetes. BMC Pregnancy Childbirth.

[49] Haase AM, Taylor AH, Fox KR, Thorp H, Lewis G (2010). Rationale and development of the physical activity counselling intervention for a pragmatic TRial of exercise and depression in the UK (TREAD-UK). Ment Health Phys Act.

[50] Carney R, Cotter J, Bradshaw T, Yung AR (2017). Examining the physical health and lifestyle of young people at ultra-high risk for psychosis: a qualitative study involving service users, parents and clinicians. Psychiatry Res.

[51] Smith CA, McNeill A, Kock L, Shahab L (2019). Exploring mental health professionals’ practice in relation to smoke-free policy within a mental health trust: a qualitative study using the COM-B model of behaviour. BMC Psychiatry.

[52] Michie S, Atkins L, West R (2014). The behaviour change wheel book - a guide to designing interventions.

[53] Myers A, Quirk H, Lowe A, Crank H, Broom D, Jones N (2021). The active hospital pilot: a qualitative study exploring the implementation of a trust-wide sport and exercise medicine-led physical activity intervention. Plos One.

[54] Cane J, O’Connor D, Michie S (2012). Validation of the theoretical domains framework for use in behaviour change and implementation research. Implement Sci.

[55] Atkins L, Francis J, Islam R (2017). A guide to using the Theoretical Domains Framework of behaviour change to investigate implementation problems. Implement Sci..

[56] Alexander KE, Brijnath B, Mazza D (2014). Barriers and enablers to delivery of the healthy kids check: an analysis informed by the theoretical domains framework and COM-B model. Implement Sci.

[57] Michie S, Richardson M, Johnston M, Abraham C, Francis J, Hardeman W (2013). The behavior change technique taxonomy (v1) of 93 hierarchically clustered techniques: building an international consensus for the reporting of behavior change interventions. Ann Behav Med.

[58] Michie S, Wood CE, Johnston M, Abraham C, Francis JJ, Hardeman W (2015). Behaviour change techniques: the development and evaluation of a taxonomic method for reporting and describing behaviour change interventions (a suite of five studies involving consensus methods, randomised controlled trials and analysis of qualitative data). Health Technol Assess (Rockv).

[59] Gould GS, Bar-Zeev Y, Bovill M, Atkins L, Gruppetta M, Clarke MJ (2017). Designing an implementation intervention with the behaviour change wheel for health provider smoking cessation care for Australian indigenous pregnant women. Implement Sci.

[60] Reid H, Caterson J, Copeland RJ. What makes a good clinical conversation on physical activity? A scoping review exploring what is known to inform the development of physical activity resources to support healthcare professionals in routine practice. OSF Prepr. 2021. 10.31219/OSF.IO/WBPXA.

[61] Michie S, Abraham C, Eccles MP, Francis JJ, Hardeman W, Johnston M (2011). Strengthening evaluation and implementation by specifying components of behaviour change interventions: a study protocol. Implement Sci.

[62] Williams SL, French DP (2011). What are the most effective intervention techniques for changing physical activity self-efficacy and physical activity behaviour - and are they the same?. Health Educ Res.

[63] Pears S, Morton K, Bijker M, Sutton S, Hardeman W (2015). Development and feasibility study of very brief interventions for physical activity in primary care. BMC Public Health.

[64] Knittle K, Nurmi J, Crutzen R, Hankonen N, Beattie M, Dombrowski SU (2018). How can interventions increase motivation for physical activity? A systematic review and meta-analysis. Health Psychol Rev.

[65] Samdal GB, Eide GE, Barth T, Williams G, Meland E (2017). Effective behaviour change techniques for physical activity and healthy eating in overweight and obese adults; systematic review and meta-regression analyses. Int J Behav Nutr Phys Act.

[66] Olander EK, Fletcher H, Williams S, Atkinson L, Turner A, French DP (2013). What are the most effective techniques in changing obese individuals’ physical activity self-efficacy and behaviour: a systematic review and meta-analysis. Int J Behav Nutr Phys Act.

[67] Howlett N, Trivedi D, Troop NA, Chater AM (2018). Are physical activity interventions for healthy inactive adults effective in promoting behavior change and maintenance, and which behavior change techniques are effective? A systematic review and meta-analysis. Transl Behav Med..

[68] Webb J, Hall J, Hall K, Fabunmi-Alade R (2016). Increasing the frequency of physical activity very brief advice by nurses to cancer patients. A mixed methods feasibility study of a training intervention. Public Health.

[69] Keyworth C, Epton T, Goldthorpe J, Calam R, Armitage CJ (2020). Delivering opportunistic behavior change interventions: a systematic review of systematic reviews. Prev Sci.

[70] Robertson R, Jochelson K (2006). Interventions that change clinician behaviour: mapping the literature.

[71] Bize R, Cornuz J, Martin B (2007). Opinions and attitudes of a sample of Swiss physicians about physical activity promotion in a primary care setting opinions and attitudes of a sample of Swiss physicians about physical activity promotion in a primary care setting. Sport Sport.

[72] Graham RC, Dugdill L, Cable NT (2005). Health professionals’ perspectives in exercise referral: implications for the referral process. Ergonomics.

[73] Abramson S, Stein J, Schaufele M, Frates E, Rogan S (2000). Personal exercise habits and counseling practices of primary care physicians: a national survey. Clin J Sport Med.

[74] Burns KJ, Camaione DN, Chatterton CT (2000). Prescription of physical activity by adult nurse practitioners: a national survey. Nurs Outlook.

[75] Sherman SE, Hershman WY (1993). Exercise counseling - how do general internists do?. J Gen Intern Med.

[76] Walsh JM, Swangard DM, Davis T, McPhee SJ (1999). Exercise counseling by primary care physicians in the era of managed care. Am J Prev Med.

[77] Goodman C, Davies SL, Dinan S, See Tai S, Iliffe S (2011). Activity promotion for community-dwelling older people: a survey of the contribution of primary care nurses. Br J Community Nurs.

[78] McDowell N, McKenna J, Naylor PJ (1997). Factors that influence practice nurses to promote physical activity. Br J Sports Med.

[79] McKenna J, Naylor PJ, McDowell N (1998). Barriers to physical activity promotion by general practitioners and practice nurses. Br J Sports Med.

[80] Steptoe A, Doherty S, Kendrick T, Rink E, Hilton S (1999). Attitudes to cardiovascular health promotion among GPs and practice nurses. Fam Pract.

[81] Kennedy MF, Meeuwisse WH (2003). Exercise counselling by family physicians in Canada. Prev Med (Baltim).

[82] Eakin EG, Smith BJ, Bauman AE (2005). Evaluating the population health impact of physical activity interventions in primary care—are we asking the right questions?. J Phys Act Health.

[83] Patel A, Schofield GM, Kolt GS, Keogh JWL (2011). General practitioners’ views and experiences of counselling for physical activity through the New Zealand Green prescription program. BMC Fam Pract.

[84] Hebert ET, Caughy MO, Shuval K (2012). Primary care providers’ perceptions of physical activity counselling in a clinical setting: a systematic review. Br J Sports Med.

[85] Yarnall KSH, Pollak KI, Østbye T, Krause KM, Michener JL (2003). Primary care: is there enough time for prevention?. Am J Public Health.

[86] Heath GW, Kolade VO, Haynes JW (2015). Exercise is MedicineTM: a pilot study linking primary care with community physical activity support. Prev Med Rep..

[87] Keyworth C, Epton T, Goldthorpe J, Calam R, Armitage CJ. Perceptions of receiving behaviour change interventions from GPs during routine consultations: A qualitative study. PLoS One. 2020;15(5):e0233399. 10.1371/journal.pone.0233399. PMID: 32437462; PMCID: PMC7241720. 10.1371/journal.pone.0233399 PMC7241720 32437462

[88] Bull FC, Schipper EC, Jamrozik K, Blanksby BA (1997). How can and do Australian doctors promote physical activity?. Prev Med (Baltim).

[89] Phillips EM (2012). A call to arms (and legs): exercise prescription for medical students. PM R.

[90] Brannan M, Bernardotto M, Clarke N, Varney J (2019). Moving healthcare professionals - a whole system approach to embed physical activity in clinical practice. BMC Med Educ..

[91] Anderson N, Ozakinci G. “It all needs to be a full jigsaw, not just bits”: exploration of healthcare professionals’ beliefs towards supported self-management for long-term conditions. BMC Psychol. 2019;7. 10.1186/s40359-019-0319-7. 10.1186/s40359-019-0319-7 PMC6591939 31234924

[92] Moser EM, Stagnaro-Green A (2009). Teaching behavior change concepts and skills during the third-year medicine clerkship. Acad Med.

[93] Chauhan BF, Jeyaraman M, Mann AS, Lys J, Skidmore B, Sibley KM, et al. Behavior change interventions and policies influencing primary healthcare professionals’ practice-an overview of reviews. Implement Sci. 2017;12. 10.1186/s13012-016-0538-8. 10.1186/s13012-016-0538-8 PMC5216570 28057024

[94] Wattanapisit A, Tuangratananon T, Thanamee S (2018). Physical activity counseling in primary care and family medicine residency training: a systematic review. BMC Med Educ.

[95] Blakeman T, Bower P, Reeves D, Chew-Graham C (2010). Bringing self-management into clinical view: a qualitative study of long-term condition management in primary care consultations. Chronic Illn.

[96] Lobelo F, Duperly J, Frank E (2009). Physical activity habits of doctors and medical students influence their counselling practices. Br J Sports Med.

[97] Lobelo F, de Quevedo IG (2014). The evidence in support of physicians and health care providers as physical activity role models. Am J Lifestyle Med.

[98] Bero LA, Grilli R, Grimshaw JM, Harvey E, Oxman AD, Thomson MA (1998). Getting research findings into practice. Closing the gap between research and practice: an overview of systematic reviews of interventions to promote the implementation of research findings. Br Med J.

[99] Yost J, Ganann R, Thompson D, Aloweni F, Newman K, Hazzan A, et al. The effectiveness of knowledge translation interventions for promoting evidence-informed decision-making among nurses in tertiary care: a systematic review and meta-analysis. Implement Sci. 2015;10:98. 10.1186/s13012-015-0286-1. PMID: 26169063; PMCID: PMC4499897. 10.1186/s13012-015-0286-1 PMC4499897 26169063

[100] Smith WR (2000). Evidence for the effectiveness of techniques to change physician behavior. Chest.

[101] Bauchner H, Simpson L, Chessare J (2001). Changing physician behaviour: editorial. Arch Dis Child.

[102] Grol R, Grimshaw J (2003). From best evidence to best practice: effective implementation of change in patients’ care. Lancet.

[103] Grimshaw JM, Thomas RE, MacLennan G, Fraser C, Ramsay CR, Vale L (2004). Effectiveness and efficiency of guideline dissemination and implementation strategies. Health Technol Assess.

[104] Crick K, Hartling L (2015). Preferences of knowledge users for two formats of summarizing results from systematic reviews: infographics and critical appraisals. Plos One.

[105] Huijg JM, van der Zouwe N, Crone MR, Verheijden MW, Middelkoop BJC, Gebhardt WA (2015). Factors influencing the introduction of physical activity interventions in primary health care: a qualitative study. Int J Behav Med.

[106] Wheeler PC, Mitchell R, Ghaly M, Buxton K. Primary care knowledge and beliefs about physical activity and health: a survey of primary healthcare team members. BJGP Open. 2017;1(2):bjgpopen17X100809. 10.3399/bjgpopen17X100809. PMID: 30564660; PMCID: PMC6169952. 10.3399/bjgpopen17X100809 PMC6169952 30564660

[107] Eakin EG, Brown WJ, Marshall AL, Mummery K, Larsen E (2004). Physical activity promotion in primary care: bridging the gap between research and practice. Am J Prev Med.

[108] Sassen B, Kok G, Vanhees L (2011). Predictors of healthcare professionals’ intention and behaviour to encourage physical activity in patients with cardiovascular risk factors. BMC Public Health.

[109] Craig P, Dieppe P, Macintyre S, Michie S, Nazareth I, Petticrew M (2006). Developing and evaluating complex interventions: new guidance prepared on behalf of the Medical Research Council by. UK.

[110] Colquhoun HL, Squires JE, Kolehmainen N, Fraser C, Grimshaw JM (2017). Methods for designing interventions to change healthcare professionals’ behaviour: a systematic review. Implement Sci.

[111] Rubak S, Sandbaek A, Lauritzen T, Christensen B (2005). Motivational interviewing: a systematic review and meta-analysis. Br J Gen Pract.

[112] Stange KC, Woolf SH, Gjeltema K (2002). One minute for prevention: the power of leveraging to fulfill the promise of health behavior counseling. Am J Prev Med.

[113] Jowsey T, Pearce-Brown C, Douglas KA, Yen L (2014). What motivates Australian health service users with chronic illness to engage in self-management behaviour?. Health Expect.

[114] Vrijens B, Vincze G, Kristanto P, Urquhart J, Burnier M (2008). Adherence to prescribed antihypertensive drug treatments: longitudinal study of electronically compiled dosing histories. BMJ.

[115] Nevins TE, Nickerson PW, Dew MA (2017). Understanding medication nonadherence after kidney transplant. J Am Soc Nephrol.

[116] Balint E (1969). The possibilities of patient-centered medicine. J R Coll Gen Pract.

[117] Grimshaw JM, Shirran L, Thomas R, Mowatt G, Fraser C, Bero L (2001). Changing provider behavior: an overview of systematic reviews of interventions. Med Care.

[118] Kennedy A, Bower P, Reeves D, Blakeman T, Bowen R, Chew-Graham C, et al. Implementation of self management support for long term conditions in routine primary care settings: cluster randomised controlled trial. BMJ. 2013;346. 10.1136/bmj.f2882. 10.1136/bmj.f2882 PMC3652644 23670660

[119] Potthoff S, Presseau J, Sniehotta FF, Johnston M, Elovainio M, Avery L. Planning to be routine: habit as a mediator of the planning-behaviour relationship in healthcare professionals. Implement Sci. 2017;12. 10.1186/s13012-017-0551-6. 10.1186/s13012-017-0551-6 PMC5319033 28222751

[120] Wood W, Quinn JM, Kashy DA (2002). Habits in everyday life: thought, emotion, and action. J Pers Soc Psychol.

[121] Grimshaw JM, Eccles MP, Lavis JN, Hill SJ, Squires JE (2012). Knowledge translation of research findings. Implement Sci.

[122] Squires JE, Sullivan K, Eccles MP, Worswick J, Grimshaw JM (2014). Are multifaceted interventions more effective than single-component interventions in changing health-care professionals’ behaviours? An overview of systematic reviews. Implement Sci.

[123] Garg AX, Adhikari NKJ, McDonald H, Rosas-Arellano MP, Devereaux PJ, Beyene J (2005). Effects of computerized clinical decision support systems on practitioner performance and patient outcomes: a systematic review. J Am Med Assoc.

[124] NICE. Behaviour change: digital and mobile health interventions. NICE guideline 183. London: National Institute for Health and Care Excellence; 2020. www.nice.org.uk/guidance/ng183.

[125] Orrow G, Kinmonth A-L, Sanderson S, Sutton S (2012). Effectiveness of physical activity promotion based in primary care: systematic review and meta-analysis of randomised controlled trials. BMJ.

